# In Memoriam

**Published:** 1879-03

**Authors:** Jas. B. Baird

**Affiliations:** Ch’m Com.


### IN MEMORIAM.

Died, at his home, in this city, November 9, 1878, Charles Rauschenberg, M.D., of congestion of the bowels, after a brief illness.

Pursuant, therefore, to the instructions of the Atlanta Academy of Medicine, the committee appointed to prepare suitable resolutions expressive of our sense of loss in the death of our esteemed friend and fellow member, beg leave to submit the following report:

*Resolved*, That in the death of Dr. Charles Rauschenberg this Academy has lost an earnest, faithful, zealous and able member.

*Resolved*, That in his life of studious devotion to the principles and practice of the great healing art, he has left to us an example worthy of imitation.

*Resolved*, That a page in the records of this Academy be dedicated to his memory, and that these resolutions be transcribed thereon.

*Resolved*, That a copy of this report be sent to the family of the deceased, and that a copy be furnished the ATLANTA MEDICAL AND SURGICAL JOURNAL for publication.

Very respectfully submitted,

JAS. B. BAIRD, M.D., Ch'm Com.

---

WE find the following in the Cincinnati *Lancet and Clinic* of January 25th : "The village of Atlanta, Georgia, finds itself possessed of more medical talent than can be accommodated in one Medical College. A charter for a new College has, consequently, been sought, and will doubtless be soon granted."

Now, the slur in the above article was evidently intended for the medical profession, but comes in very bad taste from a city dependent, in a considerable measure, upon this "*village*" for the sale of her surplus commodities. Jealousy should not rankle in the hearts of a dependent people.

